# Ultra-sensitive pressure sensing capabilities of defective one-dimensional photonic crystal

**DOI:** 10.1038/s41598-023-45680-5

**Published:** 2023-11-01

**Authors:** Arafa H. Aly, B. A. Mohamed, M. Al-Dossari, D. Mohamed, S. K. Awasthi, Mika Sillanpää

**Affiliations:** 1 TH-PPM Group, Physics Department, Faculty of Sciences, Beni-Suef University, Beni Suef, 62514 Egypt; 2 Department of Physics, Faculty of Science, King Khalid University, Abha, 62529 Saudi Arabia; 3 Department of Physics and Material Science and Engineering, Jaypee Institute of Information Technology, Noida, 201304 India; 4 Department of Chemical Engineering, School of Mining, Metallurgy and Chemical Engineering, University of Johannesburg, P. O. Box 17011, Doornfontein, 2028 South Africa; 5 Department of Biological and Chemical Engineering, Aarhus University, Nørrebrogade 44, 8000 Aarhus C, Denmark

**Keywords:** Materials science, Optics and photonics, Physics

## Abstract

Present research work deals with the extremely sensitive pressure-sensing capabilities of defective one-dimensional photonic crystal structure (GaP/SiO_2_)N/Al_2_O_3_/(GaP/SiO_2_)N. The proposed structure is realized by putting a defective layer of material Al_2_O_3_ in the middle of a structure consisting of alternating layers of GaP and SiO_2_. The transfer matrix method has been employed to examine the transmission characteristics of the proposed defective one-dimensional photonic crystal in addition to MATLAB software. An external application of the hydrostatic pressure on the proposed structure is responsible for the change in the position and intensity of defect mode inside the photonic band gap of the structure due to pressure-dependent refractive index properties of the materials being used in the design of the sructure. Additionally, the dependence of the transmission properties of the structure on other parameters like incident angle and defect layer thickness has also studied. The theoretical obtained numeric values of the quality factor and sensitivity are 17,870 and 72 nm/GPa respectively. These results are enough to support our claim that the present design can be used as an ultra-sensitive pressure sensor.

## Introduction

Pioneering research work on the photonic crystals (PhCs) was first carried out by Yablonovitch and John in 1987. They have articulated their architecture by considering a periodic arrangement of dielectric materials which may be in one-dimensional (1d), two-dimensional (2d), and three-dimensional (3d) as a 1d, 2d and 3d PhCs respectively^1,2^. The extensive research work on PhCs has been done so far. The present involvement of PhCs into variety of modern applications makes them suitable to be used as a promising candidate in research fields governing optical, chemical and medical engineering. PhCs have tremendous ability of restricting the propagation of electromagnetic waves of specific frequencies passing through them. This specific frequency range is called as photonic bandgap (PBG)^3^. The introduction of defects like cavities either by changing the thickness or material of the structure may bring sharp transmission peak inside PBG. Such modified structures are called as defective photonic crystals (DPhCs)^4^. One of the most suitable application of DPhCs is to use them as a chemical, biofluid or optical sensing structures. The easier fabrication techniques associated with 1d photonic structures allow them to be used as a most effective sensing structures due to their minute sensing and detection capabilities^5^. Many peculiar applications of 1d DPhCs related to biochemistry^6^, medical science^7–14^, petroleum engineering^9,15–19^, and other branches of sciences^20–28^ have been explored by the researchers recently. The photonic biosensors made up of 1d DPhCs have revolutionized the designing, development and testing methodology of biosensors due to significantly improved performance of such structures in terms of sensitivity (S), figure-of-merit (FoM), quality factor (Q) and detection limit (DL)^29–36^. Actually, 1d DPhCs based photonic biosensors improve the interaction time between incident photon and the target matter precured inside sensing zone of the structure for accomplishment of high-field localization. The effects of interference, phase and group birefringence associated with the biosensors composed of 2d DPHCs restrict their fabrication whereas the fascinating properties like tunable dispersion and birefringence, homogeneous and isotropic behavior of the structure and the devise compatibility makes the fabrication of 1d DPhCs biosensor easier. Such structures can be easily fabricated by adopting suitable thin film deposition techniques depending upon the choice of the materials used in the design.

The present research work is based on the photo-elastic phenomenon under the influence of the stress applied over 1d DPhC (GaP/SiO2)^N^/Al_2_O_3_/(GaP/SiO_2_)^N^. The application of stress over the structure disturbs the isotropic and homogeneous nature of the refractive indices of the contitutent materials which induces the ultra high pressure sensing capabilities into 1d DPhC composed of (GaP/SiO2)^N^/Al_2_O_3_/(GaP/SiO_2_)^N^. The architectural details along with the theoretical formulation of the structure is discussed in Section “Theoretical formulation”. Results of the work are presented in Section “Results and discussion”. Finally, conclusions are summarized in Section “Conclusion”.

## Theoretical formulation

**Figure 1:**
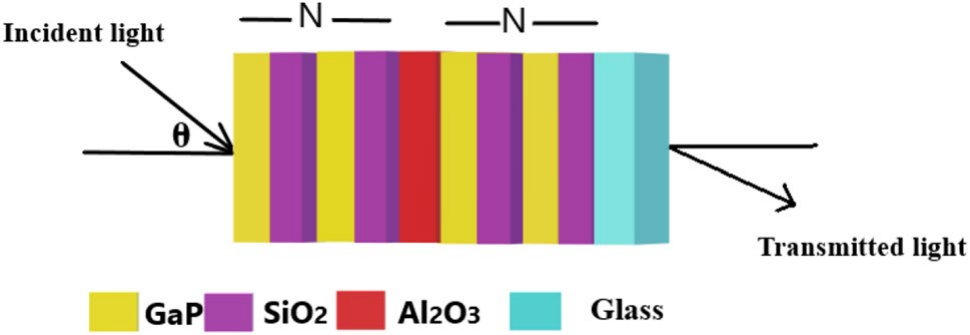
Systametic view of the propoed 1d DPhC composed of (GaP/SiO_2_)^N^ /Al_2_O_3_/(GaP/SiO_2_)^N^. The yellow, pink, red and cyan colours are representing different layers of the structure made up of GaP, SiO_2_, Al_2_O_3_ and glass materials respectively.

The transfer matrix method (TMM) has been employed to investigate the theoritical findings of the 1d DPhC composed of (GaP/SiO_2_)^*N*^/Al_2_O_3_/(GaP/SiO_2_)^*N*^ as shown in Fig. 1. The present structure can be fabricated by depositing the alternating periodic layers of gallium phosphide (GaP) and fused silica (SiO_2_) of period number *N* on either side of an additional defect layer of alluminium oxide (Al_2_O_3_) located at the middle of the structure over glass substarte. The transfer matrix approach connencting the electric and magnetic fileds of electromagnetic wave propagating into the structure near to the interface conneting every two adjesesnt layers of the structure as defined in Eq. (1) below^25–28^

1 $${\text{M}} = \left[ {\begin{array}{*{20}c} {{\text{M}}_{11} } & {{\text{M}}_{12} } \\ {{\text{M}}_{21} } & {{\text{M}}_{22} } \\ \end{array} } \right] = \left( {{\mathfrak{M}}_{1} {\mathfrak{M}}_{2} } \right)^{N} {\mathfrak{M}}_{d} \left( {{\mathfrak{M}}_{1} {\mathfrak{M}}_{2} } \right)^{N}$$

Here M_11_, M_12_, M_21_ and M_22_ are representing the elements of the resultant transfer matrix M of the structure. The symbols $\mathfrak{M}$_1_, $\mathfrak{M}$_2_ and $\mathfrak{M}$_d_ are representing the characteristic matrix of material layers GaP, SiO_2_ and Al_2_O_3_ of the structure. The subscripts 1, 2 and d are being used material layers GaP, SiO_2_ and Al_2_O_3_ respectively of the structure. The characteristic matrix $\mathfrak{M}$_n_ (*n* = 1, 2 and d) of any *n*th layer of the structure can be defined as

2 $${\mathfrak{M}}{\text{n}} = \left[ {\begin{array}{*{20}c} {\cos \left( {k_{nz} d_{n} } \right)} & {jq_{l}^{ - 1} \sin \left( {k_{nz} d_{n} } \right)} \\ {jq_{n} \sin \left( {k_{nz} d_{n} } \right)} & {\cos \left( {k_{nz} d_{n} } \right)} \\ \end{array} } \right]$$

The reflection (r) and transmission (t) coefficients of the proposed structure (GaP/SiO_2_)^*N*^/Al_2_O_3_/(GaP/SiO_2_)^*N*^ are defined by

3 $$r = \frac{{M_{21} }}{{M_{11} }},\;\;t = \frac{1}{{M_{11} }}$$

The reflectance (*R*) and transmittance (*T*) of the structure can be with the help of following relation (4)

4 $$R = \left| r \right|^{2} = \left| {\frac{{M_{21} }}{{M_{11} }}} \right|^{2} ,\;\;T = \frac{{p_{s} }}{{p_{0} }} \left| t \right|^{2} = \frac{{p_{s} }}{{p_{0} }} \left| {\frac{1}{{M_{11} }} } \right|^{2}$$

Here p_0_ = n_0_ cos(θ_0_) and p_s_ = n_s_ cos(θ_s_) are admittances of input and output ends of the structure respectively, corresponding to s-polarized wave. For p-polarized wave, input and output admittances would be p_0_ = cos(θ_0_)/n_0_ and p_s_ = cos(θ*s*)/n_s_ respectively. The symbols θ_0_ and θ_s_ are denoting angles of incidence and emergence at input and output ends of the structure respectively.

The hydrostatic pressure applied externally over the structure (GaP/SiO_2_)^*N*^/Al_2_O_3_/(GaP/SiO_2_)^*N*^ made up of acousto-optic materials can be easily measured due to change in the refractive indices of the constituent materials used in the designing of the present structure. The change in characteristics properties of the acousto-optic materials employed in the proposed pressure sensing design can be observed with the help of stress–strain formulas under the influence of externally applied pressure on the structure. This pressure over the structure is enough to affect the refractive index of the material with stress due to photo-elastic phenomenon which in turn affects the optical performance of the structure. The stress (σ) is like a pressure which determines the force acting per unit area and can be decomposed into nine components forming a secon-rank stress tensor (σ_ij)_. Here combination of the subscripts i and j is used to represesnt different combinations of x, y and z axes depending upon the direction of the force applied over the respective areas of the structure. The relation between the direction dependent refractive index (n_ij_) and the stress tensor (σ_ij_) for a cubic media can be expressed as

5 $$\left( {\begin{array}{*{20}c} {n_{xx} } \\ {\begin{array}{*{20}c} {n_{yy} } \\ {n_{zz} } \\ {\begin{array}{*{20}c} {n_{yz} } \\ {n_{xz} } \\ {n_{xy} } \\ \end{array} } \\ \end{array} } \\ \end{array} { }} \right) = \left( {\begin{array}{*{20}c} {n_{0} } \\ {\begin{array}{*{20}c} {n_{0} } \\ {n_{0} } \\ {\begin{array}{*{20}c} 0 \\ 0 \\ 0 \\ \end{array} } \\ \end{array} } \\ \end{array} { }} \right) - \left( {\begin{array}{*{20}c} {C_{1} } & {C_{2} } & {C_{2} } & 0 & 0 & 0 \\ {C_{2} } & {C_{1} } & {C_{2} } & 0 & 0 & 0 \\ {C_{2} } & {C_{2} } & {C_{1} } & 0 & 0 & 0 \\ 0 & 0 & 0 & {C_{3} } & 0 & 0 \\ 0 & 0 & 0 & 0 & {C_{3} } & 0 \\ 0 & 0 & 0 & 0 & 0 & {C_{3} } \\ \end{array} } \right)\left( {\begin{array}{*{20}c} {\sigma_{xx} } \\ {\begin{array}{*{20}c} {\sigma_{yy} } \\ {\sigma_{zz} } \\ {\begin{array}{*{20}c} {\sigma_{yz} } \\ {\sigma_{xz} } \\ {\sigma_{xy} } \\ \end{array} } \\ \end{array} } \\ \end{array} { }} \right)$$

The *σ*_ij_ is the stress applied over any *ij* plane of a cubic crystal resulting a corresponding change in the refractive index of the material denoted as *n*_ij_. If the pressure applied on the structure from only one direction with respect to normal on the plane of the structure, the whole device is said to under stress. In this condition, ${\sigma }_{xx}$ = ${\sigma }_{yy}$ = ${\sigma }_{zz}$  = σ and ${\sigma }_{xy}$ = ${\sigma }_{yz}$ = ${\sigma }_{zx}$  = 0. Under this condition the stress state is said to be in-plan due to stress anisotropy effect resulting a refractive index of the medium as $n={n}_{0}-({C}_{11}+2{C}_{12})\sigma$. Here *n*_0_ is the refractive index in absence of stress.

The stress-optic constants, *C*_*k*_ (*k* = 1, 2, 3) used in the above relation (5) are defined as

6 $$C_{1} = \frac{{n_{0}^{3} \left( {p_{11} - 2Vp_{12} } \right)}}{2E},\;\,C_{1} = \frac{{n_{0}^{3} \left( {p_{11} - 2Vp_{12} } \right)}}{2E},\;\;{\text{and}}\;\;C_{3} = \frac{{n_{0}^{3} p_{44} }}{2G}$$

Here alphabets E, G, and V are representing young’s modulus, shear modulus, and Poisson’s ratio respectively. The strain-optic constants are being shown by notations *p*_11_, *p*_12_ and *p*_44_. For isotropic crystals they are mutually connected as

7 $$p_{44} = \frac{{\left( {p_{11} - p_{12} } \right)}}{2},\;\;G = \frac{E}{{2\left( {1 + V} \right)}}$$

## Results and discussion

This section deals with the performance of the suggested pressure sensing photonic structure which is the main objective of the work. The thickness of alternating periodic layers of materials GaP and SiO_2_ of the proposed structure 115 nm and 273 nm respectively. The thickness of the defect layer of material Al_2_O_3_ is 255 nm located at middle of the structure shown in Fig. 1. All the layers of the proposed pressure sensing structure is made up of elasto-optic materials GaP, SiO_2_ and Al_2_O_3_. The period number N of the structure has been fixed to 5. The numeric values of stress-optic (*C*_k_) and strain-optic (*p*_ij_) constants associated with the materials GaP, SiO_2_ and Al_2_O_3_ are given in Table 1 below.

**Table 1:** The numeric values of photo-elastic constants of materials GaP, SiO_2_ and Al_2_O_3_ calculated at free space wavelength λ_0_ = 633 nm.

Materials	*n*_0_	*p*_11_	*p*_12_	*p*_44_	*C*_1_ (10^–12^/Pa)	*C*_2_ (10^–12^/Pa)	*C*_3_ (10^–12^/Pa)
GaP	3.32	− 0.151	− 0.082	− 0.074	− 17.91	− 1.87	− 19.21
SiO_2_	1.46	0.121	0.270	–−	0.65	4.50	− 3.85
Al_2_O_3_	1.76	− 0.23	− 0.03	− 0.10	− 1.61	0.202	− 1.90

**Figure 2:**
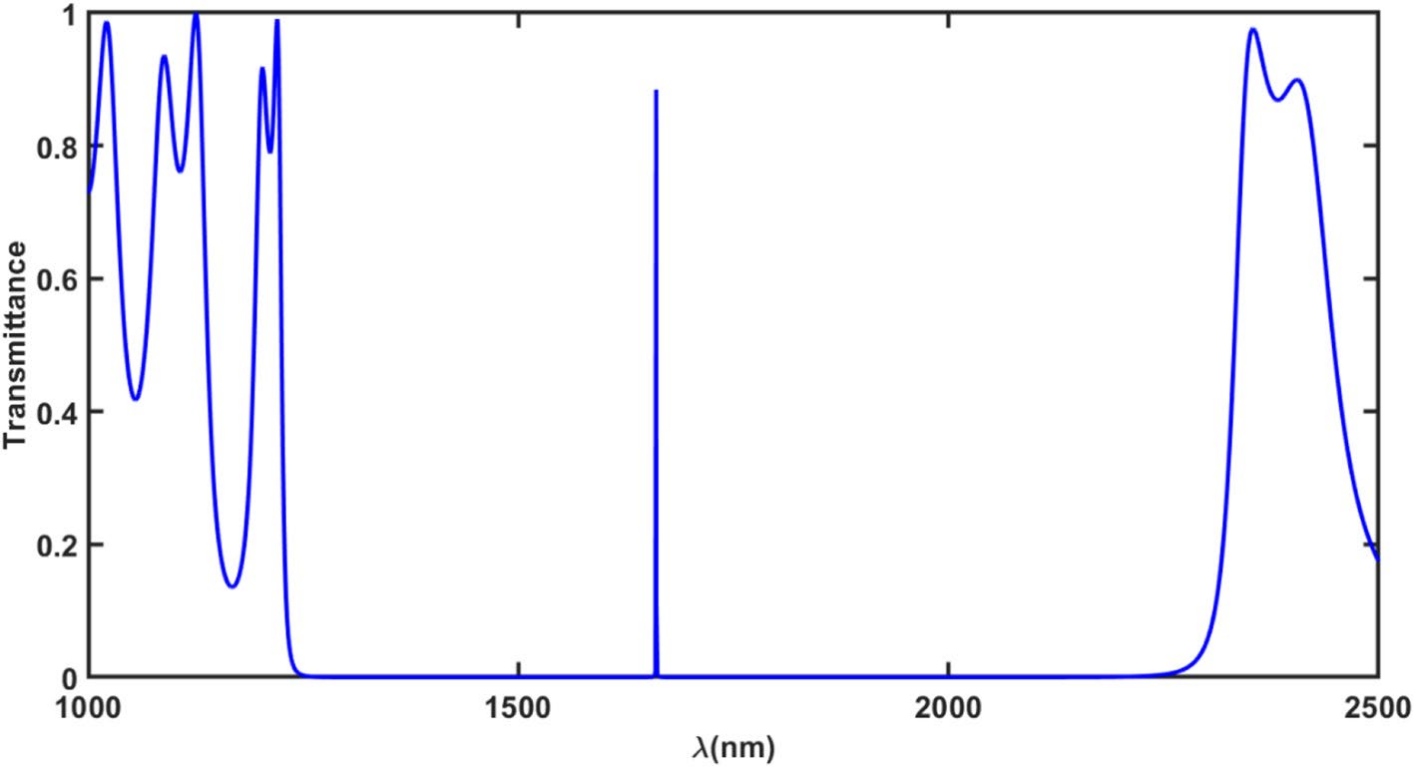
Transmittance of proposed structure (GaP/SiO_2_)^N^ /Al_2_O_3_/(GaP/SiO_2_)^N^ under normal incidence in absence of an external pressure (0 GPa).

First transmission spectra under normal incidence in absence of an external pressure has been computed by using MATLAB programing in addition to TMM as shown in Fig. 2. It exhibits a single defect mode of transmittance 88% located at 1660.24 nm inside PBG extending from 1281.6 to 2244.6 nm as shown in Fig. 2. This defect mode arises inside PBG of the structure due to insertion of an additional layer of Al_2_O_3_ of thickness 255 nm which disturbs the periodicity of the structure. Next efforts have been given to analyze the effect of pressure applied externally on the structure. This analysis makes us aware about the pressure sensing capabilities of our structure due to presence of elasto-optic materials in the design.

### Effect of pressure on the performance of the proposed sensor

**Figure 3:**
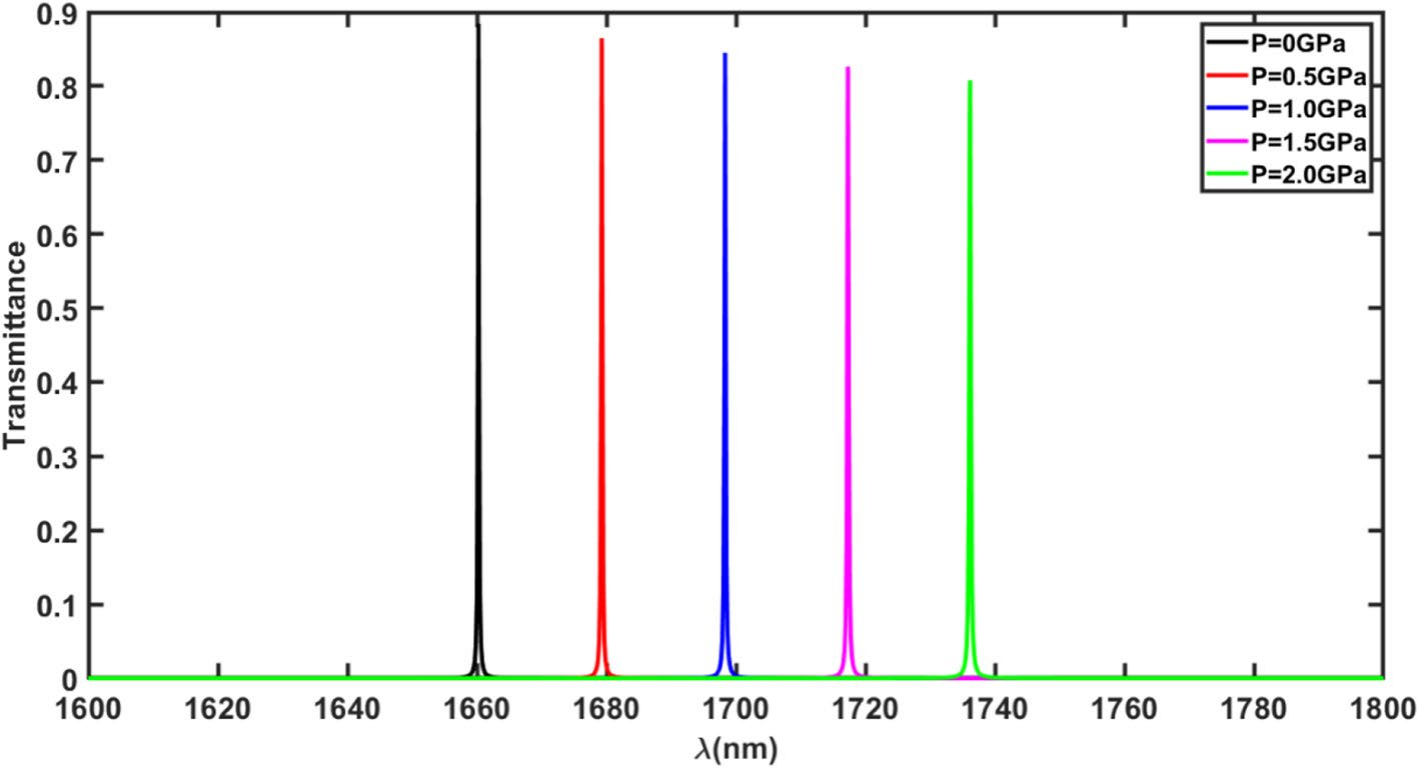
Transmittance of proposed structure (GaP/SiO_2_)^N^ /Al_2_O_3_/(GaP/SiO_2_)^N^ under normal incidence corresponding to external pressure. The transmittance peaks of colours black, red, blue, pink and green are corresponding to external pressure 0, 0.5, 1.0, 1.5 and 2.0 GPa respectively.

In this section, we have examined how the position and intensity of the defect mode inside PBG of the proposed 1d DPhC (GaP/SiO_2_)^N^ /Al_2_O_3_/(GaP/SiO_2_)^N^ is affacted under the influence of an external pressure which varies from 0 GPa to 2.0 GPa in steps of 0.5 GPa. This purpose is acommplished by applying the pressure 0.5, 1.0, 1.5 and 2.0 GPa seperalely on the structure along the priodicity of the structure and obserbed the respective changes on the transmission spectra of structure as shown in Fig. 3. It shows five transmission peaks of black, red, blue, pink and green colours inside PBG of the structure corresponding to externally applied pressure of values 0, 0.5, 1.0, 1.5 and 2.0 GPa respectively. One can easily observe that the defect mode is started to shift towards higher wavelength side as the pressure increases. The maximum shifting in the position of defect mode is observed corresponding to pressure 2.0 GPa. Though the application of an external pressure on the structure reduces the intensity of the defect mode inside PBG of the proposed structure from 88 to 80.79% as the pressure increases from 0 to 2.0 GPa. This reduction in the intensity of the defect mode inside PBG due to incerese in pressure is sufficient to be examined by the optical spectrum analyzer available for the purpose. Actually, application of external pressure on the structure increases the refractive index of the defect layer made up of material Al_2_O_3_ due to elasto-optic effect (Table 2) responsible for the shifting of defect mode inside PBG. Moreover external pressure on the structure creates the pressure dependent imperfactions in the material which are responsible for scattering losses which results decrease in the intensity of the defect mode inside PBG. These imperfactions reach to maximum under the pressure 2.0 GPa resulting the maximum drop in the intensity of defect mode as shown in Fig. 3.

**Table 2:** Externally applied pressure dependent refractive index values of material Al_2_O_3_.

Applied pressure (GPa)	Refractive index (RIU)
0	1.76
0.5	2.36
1.0	2.97
1.5	3.57
2.0	4.18

**Figure 4:**
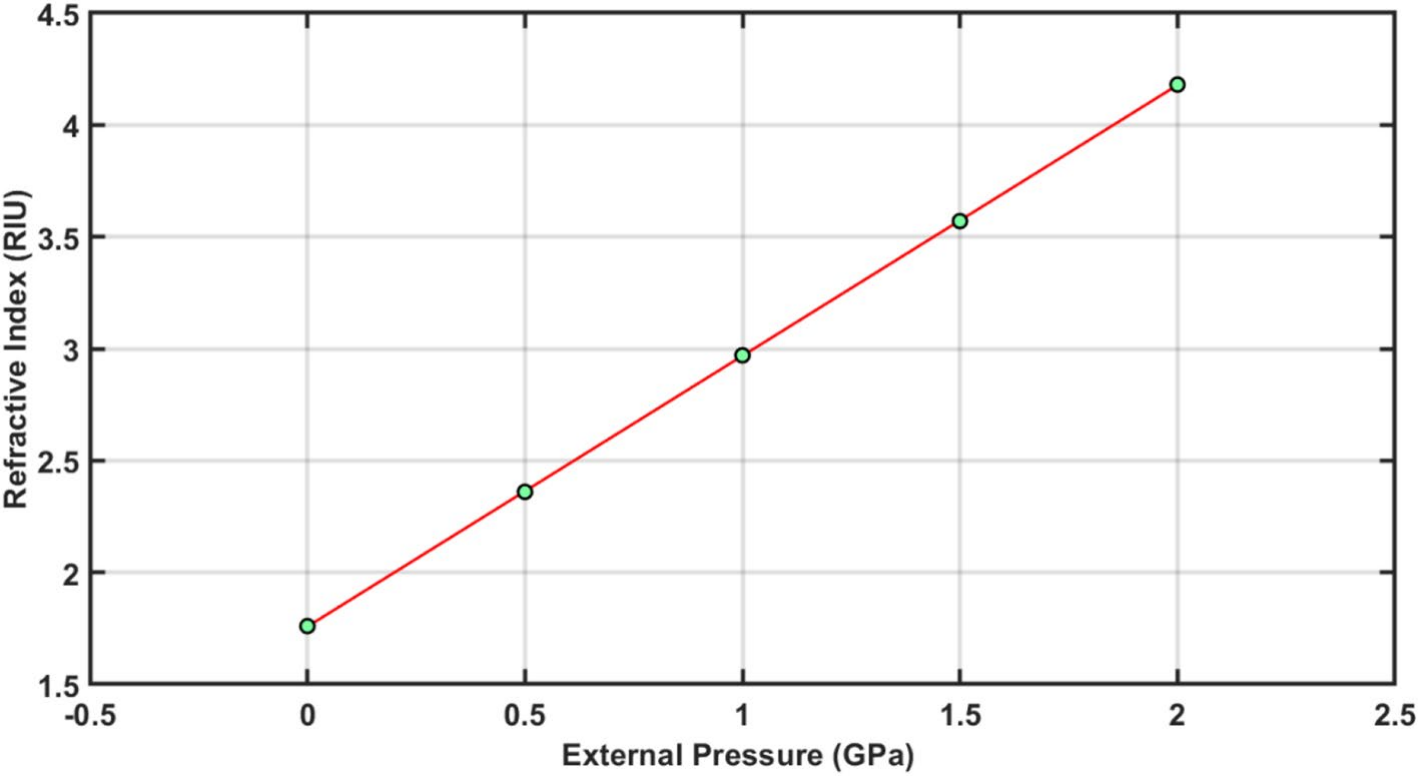
External pressure dependent refractive index values of Al_2_O_3_ are shown by green colour solid balls and red colour solid line is showing the linear curve fitting applied over the data.

The linear curve fitting has been applied on to data presented in Table 2 for getting pictorial representation. The curve fitting results are shown in Fig. 4 below. It shows the perfect linear relationship between the external pressure dependent refractive index values of Al_2_O_3_ material layer responsible for creating defect. Aforementioned linear shifting of refractive index (*n*_*d*_) of Al_2_O_3_ dependent upon external pressure (*P*) is fitted with following curve fitting as

8 $$P = 1.21n_{d} + 1.758,\;\;\left( {R^{{2}} = {1}} \right)$$

Here *R*^2^ is the corellation factor which high as expected.

### Effect of defect layer thickness on the performance of the proposed sensor

Next, efforts have been further extended to examine the pressure sensing capabilities of different pressure sensors of defect layer thicknesses dd = 100 nm, dd = 200 nm, dd = 300 nm and dd = 400 nm, each under the influence of external pressure 0.5 GPa, 1.0 GPa, 1.5 GPa and 2.0 GPa with respect to absence of an external pressure applied over the structures. The purpose of this additional work is to identify the most sensitive structure amongst all the pressure sensing 1d DPhC structure considered in this study. This time we have calculated the sensitivity of each structures of defect layer thickness dd = 100 nm, dd = 200 nm, dd = 300 nm and dd = 400 nm under the influence of external pressure 0.5, 1.0, 1.5 and 2.0 GPa with respect to absence of an external pressure applied over the structures. The sensitivity (S) of the pressure sensor is one of the most significant parameters which determines how minutely and accurately our sensor can sense the change in the defect mode position (Δ*λ*) inside PBG of the structure with respect to change in the external pressure (Δ*P*) applied over the structure. For our structure sensitivity can be defined as

9 $$S = \frac{{{\Delta }\lambda }}{{{\Delta }P}}\;\left( {{\text{nm}}/{\text{GPa}}} \right)$$

**Figure 5:**
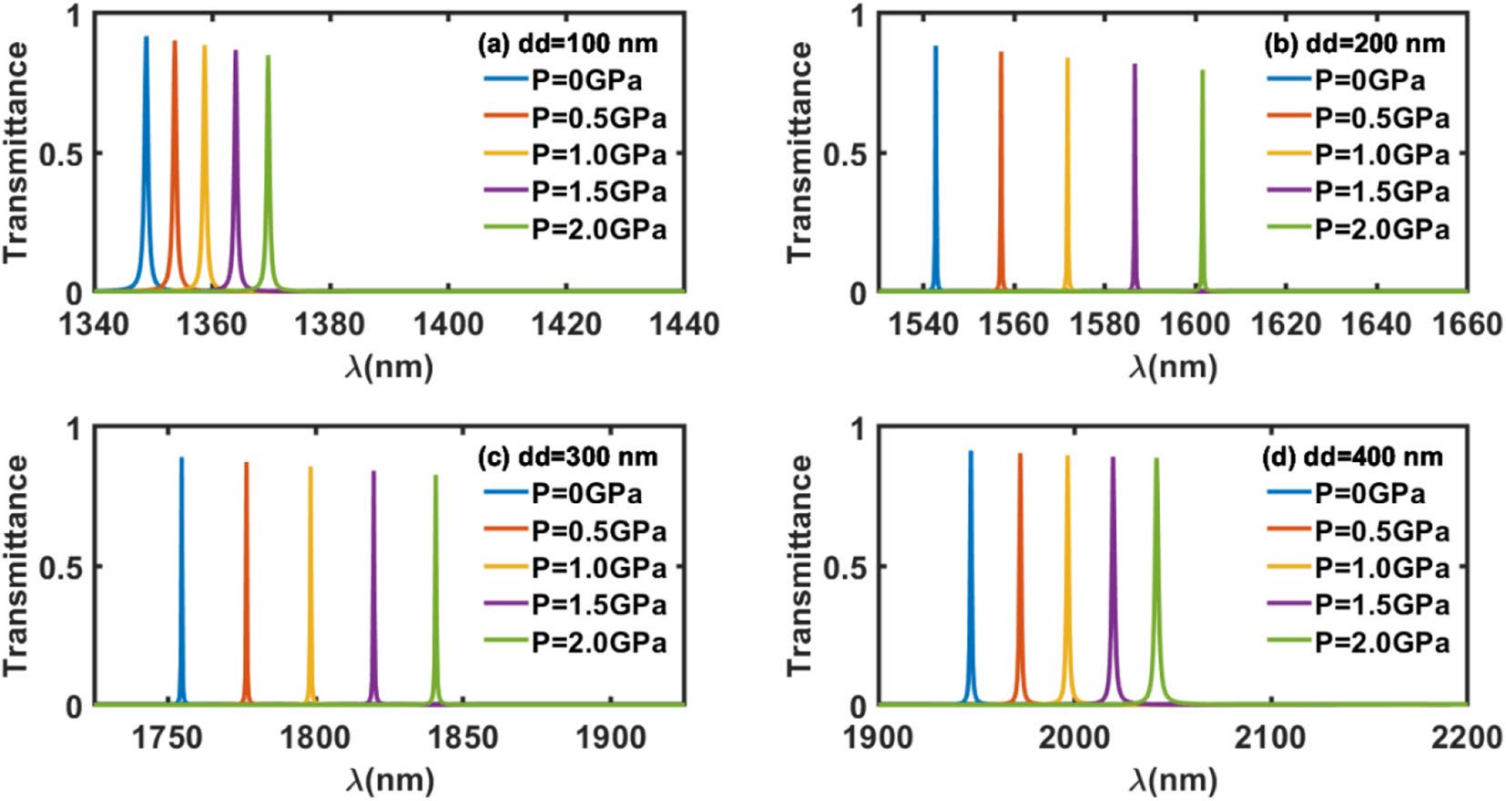
Transmittance of proposed structure (GaP/SiO_2_)^N^/Al_2_O_3_/(GaP/SiO_2_)^N^ of defect layer thickness (**a**) dd = 100 nm, (**b**) dd = 200 nm, (**c**) dd = 300 nm and (**d**) dd = 400 nm, under normal incidence corresponding to different external pressures. The transmittance peaks of colours blue, red, yellow, violet and green are corresponding to external pressure 0 GPa, 0.5, 1.0, 1.5 and 2.0 GPa respectively.

Next, transmittance of all four pressure sensors of defect layer thickness 100, 200, 300 and 400 nm, each under the influence of external pressure 0, 0.5, 1.0, 1.5 and 2.0 GPa have been plotted separately in Fig. 5 below. These transmittance plots are the mandatory requirement to calculate the sensitivity of each structures under the influence of an external pressure. It is evident from Fig. 5 that as the thickness of defect layer of each structure increases from 100 to 400 nm, pressure dependent defect modes inside PBGs of the structures of defect layer thickness 100, 200, 300 and 400 nm shown in blue, red, yellow, violet and green colours corresponding to external pressure 0, 0.5, 1.0, 1.5 and 2.0 GPa respectively have red shift. Moreover, their full width half maximum (FWHM) also improves which is an indicator for identifying the most sensitivity structures. Any increase in the thickness of defect layer beyond 400 nm pushes the pressure dependent defect modes outside the PBG extending from 1281.6 to 2244.6  nm as shown in Fig. 2 which is against the working principle of the photonic biosensors.

The sensitivity of all four structures of defect layer thickness 100, 200, 300 and 400 nm under the influence of fixed external pressure 0.5 GPa has been calculated by using Eq. (9) with the help of Fig. 5a, b, c and d respectively. The sensitivity values of all four structures are listed in Table 3 below.

**Table 3:** Sensitivity of photonic pressure sensing structures of different defect layer thickness under the influence fixed external pressure 0.5 GPa.

Defect layer thickness (nm)	Sensitivity (nm/GPa)
100	10
200	28
300	44
400	50

**Figure 6:**
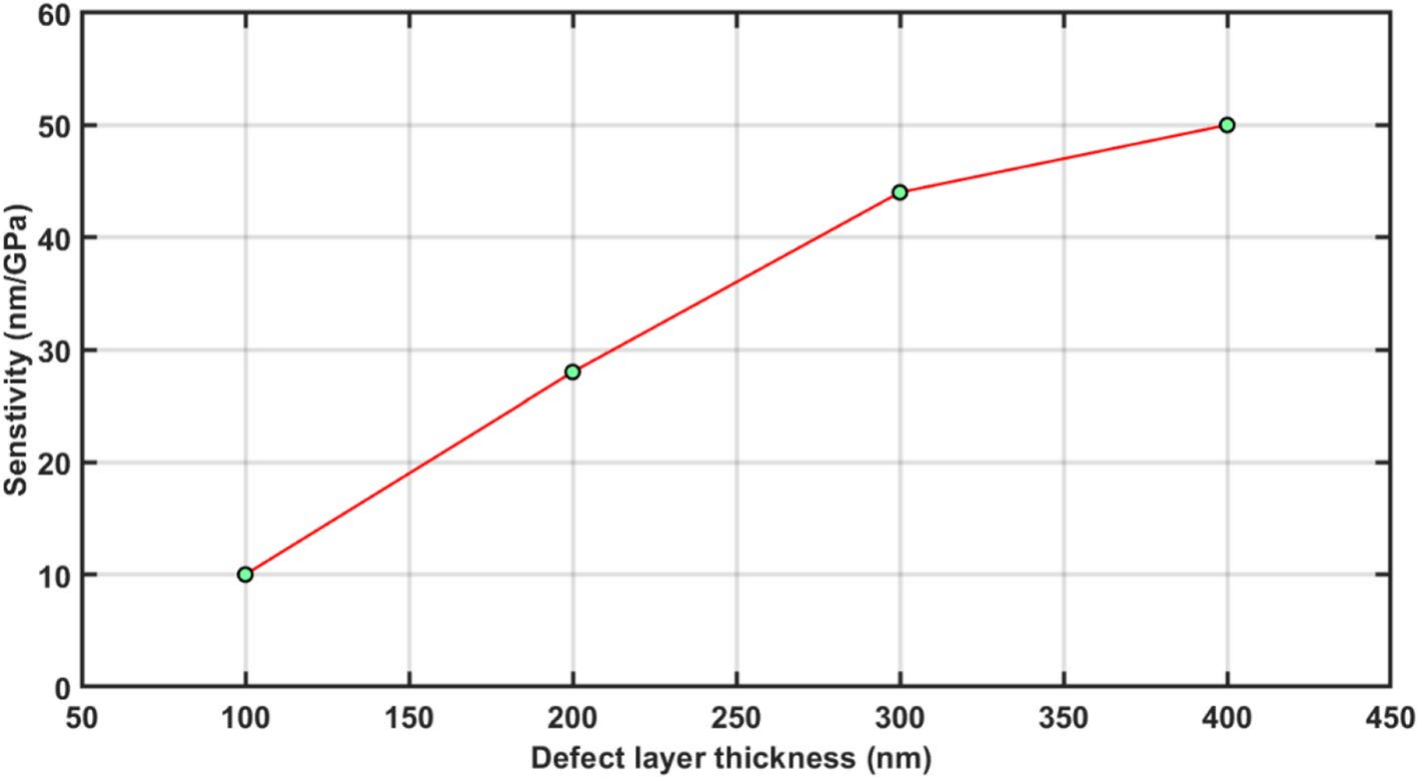
A direct relationship between the sensitivity and defect layer thickness of the structures under the influence of fixed external pressure of 0.5 GPa.

The sensitivity calculated data presented in the Table 3 shows that the 1d DPhC with defect layer thickness 400 nm is the most sensitivity pressure sensing structure which gives a sensitivity of 50 nm/GPa. The numeric data giving defect layer thickness dependent sensitivity of the structure under the influence of fixed external pressure of 0.5 GPa as mentioned in Table 3 has been plotted in Fig. 6 below. It shows that the linear relationship between defect layer thickness of the structures and their sensitivity values for the structure with dd = 100, 200 and 300 nm. But for the structure with dd = 400 nm the restricted increase in the sensitivity of 50 nm/GPa is noticed which reaches to semi-steady state.

### Effect of angle of incidence on the performance of the proposed sensor

**Figure 7:**
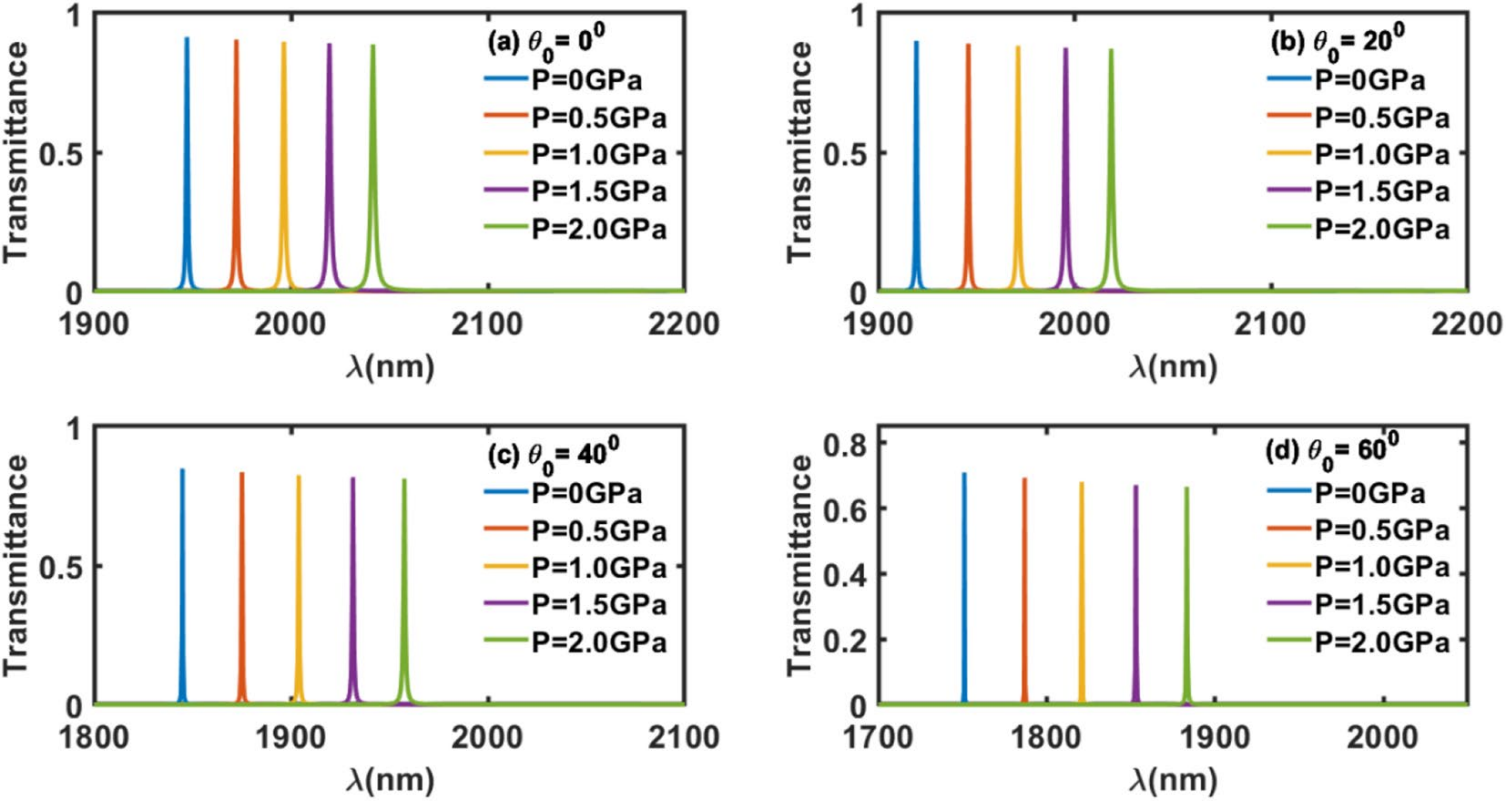
Transmittance of proposed structure (GaP/SiO_2_)^N^ /Al_2_O_3_/(GaP/SiO_2_)^N^ of defect layer thickness dd = 400 nm with (**a**) θ_0_ = 0°, (**b**) θ_0_ = 20°, (**c**) θ_0_ = 40° and (**d**) θ_0_ = 60° corresponding to different external pressures. The transmittance peaks of colours blue, red, yellow, violet and green are corresponding to external pressure 0, 0.5, 1.0, 1.5 and 2.0 GPa respectively.

After getting optimized thickness of the defect layer dd = 400 nm to design a pressure sesnor of maximum sesntivity of 50 nm/GPa under normal incidence, we have focused ourself to study the effect of change in angle of incidence (θ_0_) from normal incidence (θ_0_ = 0°) to oblique incidence (θ_0_ ≠ 0°) and observe the respective change on the senstivity of the 1d DPhC with optimized defect layer thickness dd = 400 nm. For this purpose the transmittance spectra of the photonic pressure sesning structure (GaP/SiO_2_)^N^/Al_2_O_3_/(GaP/SiO_2_)^N^ with dd = 400 nm have been recorded at different incident angles θ_0_ = 0°, 20°, 40° and 60° as shown in Fig. 7a, b, c and d respectively. It has been noticed in Fig. 7 that as angle of incidence approaches from normal to oblique incidence condition, the externally applied pressure dependent defect modes inside PBG of the structure have blue shift with reduction in the intensity of the defect modes. This reduction in the intensity of the defect mode is due to enhancement in the reflectivity of the structure under oblique incidence. The condition of oblique incidence reduces the FWHM of each defect modes both in absence and presence of an external pressure applied over the structure. This reduction in the FWHM of the pressure influenced defect modes is more meaning full as compare to reduction in the intensity. The reduction in the intensity of the defect mode is from 91 to 71% as *θ*_0_ changes from 0 to 60° respectively under external pressure of 0.5 GPa as evident from Fig. 7. Moreover this reduction in the intensity is well above the threshhold value of the energy requirement of the electronic detectors used for the purpose.

The information obtained from Fig. 7 has been utilized for calculating sesntivity of the structure with dd = 400 nm at θ_0_ = 0°, 20°, 40° and 60° with the help of Eq. (9) corresponding to external pressure of 0.5 GPa. The numeric values of the sesntivity of the structure with dd = 400 nm at θ_0_ = 0°, 20°, 40° and 60° under the influence of an external pressure 0.5 GPa are being summarized in Table 4. Form the data of the Table 4 one can easily see the effect of changing the angle of incidence from normal incidence to oblique incidence. As θ_0_ increases from 0°, the sesntivity of the proposed pressure sesning structure with optimized defect layer thickness dd = 400 nm increases. This increase in sesntivity is smaller under lower incident angles but significantely improves to 72 nm/GPa at θ_0_ = 60°. Any increase in the θ_0_ beyond 60° resullts the drastic fall in the intensity of defect mode with and with out external pressure. This major reduction in the intensity of the defect mode is below the threshhold value of the energy requirement of the electronic detectors used for the display the results of the sesnor.

**Table 4:** Angle of incidence dependent sensitivity of photonic pressure sensing structure with dd = 400 nm under the influence fixed external pressure 0.5 GPa.

Angle of incidence (in degree)	Sensitivity (nm/GPa)
0°	50
20°	54
40°	60
60°	72

**Figure 8:**
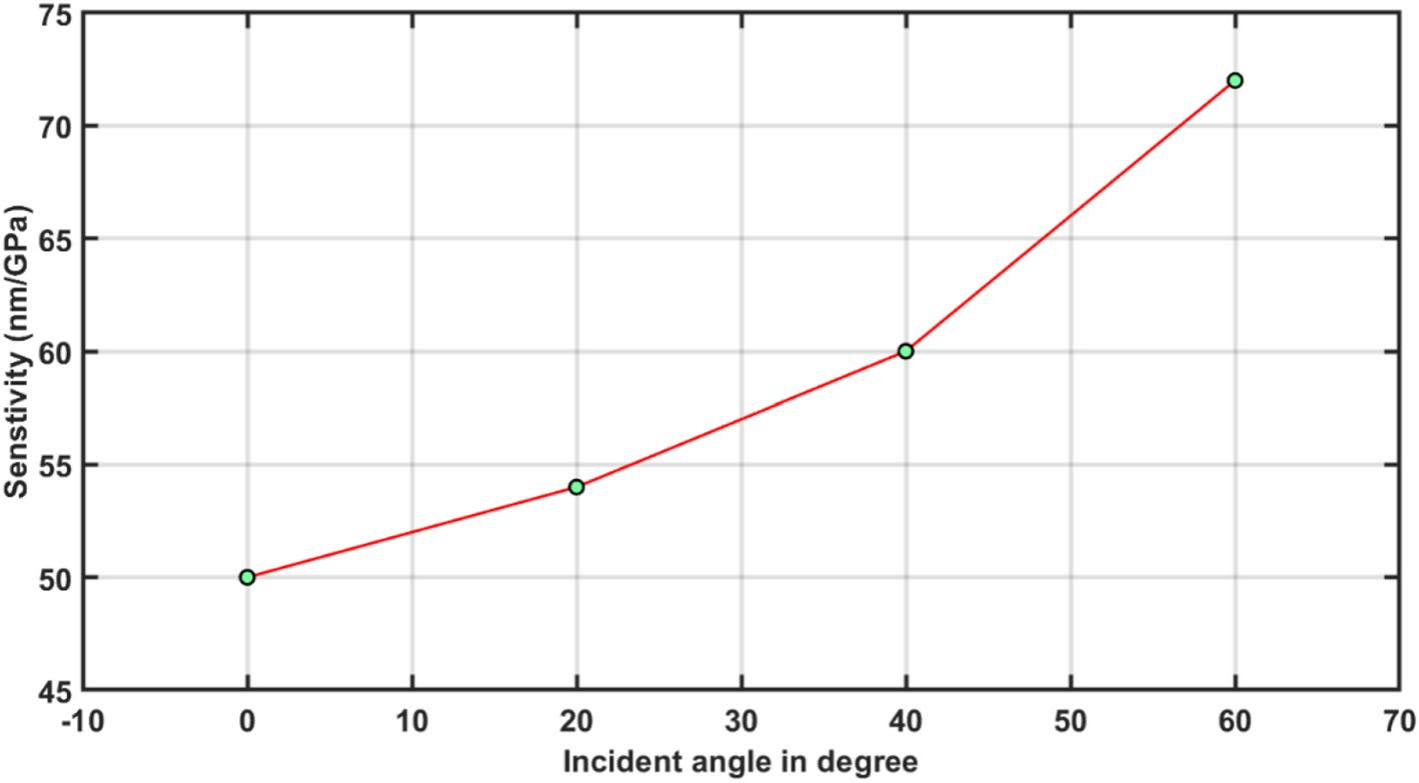
A direct relationship between the sensitivity and incident angle of the structures with dd = 400 nm under the influence of fixed external pressure of 0.5 GPa.

The results of the Table 4 are plotted in Fig. 8 to observe the impact of incident angle on the sensitivity of the proposed 1d photonic pressuring sensing design with optimized defect layer thickness *dd* = 400 nm under the influence of an external pressure 0.5 GPa. It shows that under lower incident angles sensitivity increases nearly linear. Under higher incident angles the various of sensitivity does not follow linear relationship. Moreover, the nature of the sensitivity variations with respect to defect layer thickness and incident angles are opposite in nature as evident from Figs. 6 and 8 respectively because of their respective redshift and blueshift variations of the transmission spectra as shown in Figs. 5 and 7.

Finally, we have examined the performance of our 1D photonic pressure sesning structure (GaP/SiO_2_)^N^/Al_2_O_3_/(GaP/SiO_2_)^N^ with optimized defect layer thickness dd = 400 nm at θ_0_ = 60°. This perofrmance evaluation has been done on the basis of three additional parameterts like quality factor (*Q*_f_), figure of merit (FoM) and signal-to-noise ratio (SNR) as defined in refences [X–Y]. The numeric values of *Q*_f_, FoM and SNR in addition to the sensitivity have been summarized in Table 5 below.

**Table 5:** Evaluation of the performance of optimized photonic pressure sesning structure on the basis of S, Q_f_, FoM and SNR at θ_0_ = 60°.

Pressure (GPa)	Refractive index (RIU)	*λ* (nm)	*S* (nm/GPa)	FWHM (nm)	Q_f_	FoM	SNR
0	1.76	1751	–-	0.060	29,183.3	–	–
0.5	2.36	1787	72	0.100	17,870	0.004	360
1.0	2.97	1821	70	0.125	14,568	0.0048	560
1.5	3.57	1853	68	0.175	10,588.6	0.0064	582.8
2.0	4.18	1883	66	0.225	8368.8	0.0078	586.6

From the data of Table 5, one can see that the application of the external pressure on the structure displaces the central wavelength of defect mode towards higher wavelength side. The maximum displacement is corresponding to external pressure of 2.0 GPa. The sensitivity of structure reduces slightly from 72 to 66 GPa as external pressure increases from 0.5 to 2.0 GPa respectively. This variation also improves the FWHM from 0.060 to 0.225 nm. The quality factor of the proposed structure remains high as expected though it reduces as pressure increases. The figure of merit of our pressure sensor remains low and study. The signal to noise ratio values of the proposed pressure sensor increases as we apply external pressure and becomes maximum at 2.0 PGa which is another indicator showing the minute pressure sensing.

The performance of proposed pressure sensor composed of 1d DPhC has been compared with the similar kind of work carried out by various eminent photonic worker between 2012 and 2020. The comparison based on sensitivity amongst the present and past reported works has been listed in Table 6 below. This comparison helps us to infer that the proposed pressure sensor composed of (GaP/SiO_2_)^5^/Al_2_O_3_/(GaP/SiO_2_)^5^ with dd = 400 nm and θ_0_ = 60° shows extremely high sensitivity value of 72 nm/PGa when the externally applied pressure on the design is 0.5 (GPa). The sensitivity remains almost constant under the application of extremely high pressure of 2.0 GPa which makes our design special and useful as compared to their counterparts.

**Table 6:** Comparison of the proposed sensor design with the recent works.

References	Sensitivity (nm/GPa)	Year
Ref^37^	8.6	2020
Ref^38^	2	2014
Ref^7^	8	2012
Ref^39^	13.9	2016
Proposed sensor	72	–

## Conclusion

In conclusion, we have theoretically examined a very sensitive pressure sensor based on a 1d photonic crystal with a defect. The suggested 1d photonic structure can be used as a sensitive hydrostatic pressure sensor due to their capability of detecting a very little change in the refractive index of the acousto-optic materials used in the design under the influence of an external pressure. In this work effect of various internal and external parameters on the device performance have been studied. Our design possesses the high-quality factor value of 17,870, and maximum sensitivity of 72 nm/GPa under the externally applied pressure of 0.5 GPa. The results of this study can be utilized as a framework of constructing a top-notch pressure sensor.

## Data Availability

The data that support the findings of this study are available from the corresponding author upon reason-able request.
